# The Bioactive Potential of Fruit Juice of Black Chokeberry (*Aronia melanocarpa*) Produced in Edirne Province‐Türkiye: Phenolic Profile, Elemental Composition, and In Vitro Antioxidant and Antibacterial Activities

**DOI:** 10.1002/fsn3.70784

**Published:** 2025-08-26

**Authors:** Fatmagul Halici Demir, Gokhan Acik, Duygu Sahin Gul

**Affiliations:** ^1^ Department of Food Processing, Arda Vocational School Trakya University Edirne Turkey; ^2^ Department of Chemistry and Chemical Processing Technologies, Arda Vocational School Trakya University Edirne Turkey; ^3^ Chemistry and Chemical Processing Technologies Trakya University Edirne Turkey

**Keywords:** antibacterial, antioxidant, *Aronia melanocarpa*, flavonoids, polyphenols

## Abstract

In today's world, the market and consumption of fruits are constantly increasing due to the versatility offered by the presentation of fruits in many different forms when marketed. In particular, the nutritional properties of fruits and the types that provide benefits such as antioxidant, antimicrobial, and anti‐inflammatory properties also positively affect this situation. The term ‘superfruit’ has been coined to highlight the exceptional nutritional value and distinctive health‐promoting phytochemicals of specific fruits. The present study purposed therefore to systematically explore the phenolic profile, macro/micro‐element ingredients, and the capacity of in vitro antioxidant and antibacterial activity in fruit juice from *Aronia melanocarpa* , a fruit widely recognized as a superfruit, cultivated for four years in Edirne Province, Türkiye. *Aronia melanocarpa* fruit juice (AFJ) used in our experiment was rich in phenolic acid (2.37 ± 0.28 mg gallic acid equivalents mL^−1^ juice) and flavonoid substances (4.68 ± 0.45 mg catechin equivalents mL^−1^ juice). Considering the effect of both AFJ concentration and treatment duration, the higher antioxidant activity was observed in ABTS^+•^ assay compared with the DPPH^•^ assay. The IC_50_ of DPPH^•^ and ABTS^+•^ scavenging abilities were 26.31 ± 0.65 and 27.99 ± 0.78 μL mL^−1^, respectively, and the MIC of AFJ against Gram‐positive and Gram‐negative bacteria were 71,687 and 286,750 μg mL^−1^, respectively. Aronia juice from Edirne, rich in polyphenolic substances, has the potential to be used directly/indirectly as both an antioxidative and antibacterial additive in the production of functional beverages or foods.

## Graphical abstract

The study investigates the bioactive properties of *Aronia melanocarpa* (black chokeberry) juice produced in Edirne, Türkiye. It found that the juice is rich in phenolic acids and flavonoids, showing significant antioxidant and antibacterial activity, especially against Gram‐positive bacteria. These findings suggest its potential use in functional foods and beverages as a natural health‐promoting additive.

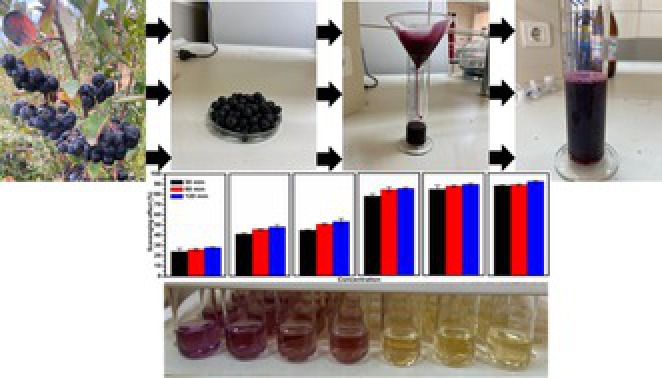

## 1 Introduction

In recent years, plant‐derived antioxidants have garnered growing interest among food scientists, manufacturers, and consumers alike. Many types of vegetables and fruits, berries, leaves, cereals, nuts, tree materials, seeds, spices, etc. have been investigated as potential sources of phenolic compounds with antioxidative activity (Hu et al. 2022; Lazaridis et al. 2024; Lu et al. 2011). The antioxidant activity of phenolic compounds is mainly due to their reducing, hydrogen donor, and singlet oxygen quenching properties (Angelé‐Martínez et al. 2022). In the literature, studies have found that flavonoids and other phenolics have a preventive effect on the progression of extremely serious diseases such as cancer and heart disease (Pap et al. 2021). The main flavonoid derivatives in berries are anthocyanins, proanthocyanins, flavonols, and catechins, while phenolic acids are hydroxyl‐functionalized species of cinnamic acid and benzoic acid (Macheix et al. 1990).

The recognized health benefits of berry‐derived antioxidants have increasingly attracted the attention of researchers, food producers, and consumers toward fruits with functional health properties. Berries are very rich in phenolic compound content such as flavonoids and phenolic acids, which exhibit antioxidant activity. In this respect, historically, dark‐colored berries, similar in size and color to blackcurrants, have been used in traditional medicine to treat colds or directly as a food source (Rousseau 1947).

In a study conducted by Kähkönen et al. (1999), it was revealed that the total phenolic content of the berries was more than 20 mg g^−1^ (gallic acid equivalents). On the other hand, the dark red color of these berries primarily results from anthocyanins such as cyanidin 3‐*O*‐galactoside, 3‐*O*‐arabinoside, 3‐*O*‐glucoside, and 3‐*O*‐xyloside (Wang et al. 2023). As aromatic acids, chlorogenic and neochlorogenic acids are prominent, and the high phenolic content detected in these berries is directly proportional to antioxidant activity (Ga̧siorowski et al. 1997; Jurikova et al. 2017).

Studies conducted to determine the antioxidant activity of fruit extracts have shown that fresh strawberry extract has a much more advanced antioxidant capacity than trolox in an artificial peroxyl radical model system (Dzhanfezova et al. 2020). Moreover, raspberry, blackberry, black and red currant, and blueberry‐based extracts have also been found to have highly effective activity against chemically generated radicals, as well as inhibiting human low density lipoprotein (LDL) and liposome oxidation (Kocabas and Sanlier 2024; Zorzi et al. 2020).

Black chokeberry or aronia, *Aronia melanocarpa* (hereinafter referred to as ‘aronia’), is a member of the Rosaceae family and is known as a native shrub found especially in eastern parts of North America (Jeppsson 1999). It was introduced into Europe in the early twentieth century and has since been widely cultivated all over the world (Gurčík et al. 2023). During the last two or three decades, aronia has been the subject of scientific studies as both a valuable source of important phytonutrients (Sosnowska et al. 2022) and a potential food colorant (Bridle and Timberlake 1997). Meanwhile, high antioxidant properties have been reported for aronia due to its high phenolic content and even better than blueberry or cranberry in terms of the previously mentioned superior properties. The astringent effect in its taste clearly proves the presence of tannin content in its structure, and this content is higher than that of other fruits. On the other hand, in addition to numerous studies on the antioxidant and anti‐aging activities of aronia, it cannot be ignored that it has many health effects, particularly such as antibacterial, anti‐inflammatory, anticancer, antidepressant, and anti‐fatigue (Jurikova et al. 2017; Raudsepp et al. 2019; Shi et al. 2024; Sidor et al. 2019; Tomić et al. 2016).

In summary, the determination and characterization of flavonoids and phenolic compounds, as well as their extraction and purification, are important for the evaluation and development of aronia resources. Pure or processed/functionalized aronia has many advantages that need to be considered and evaluated in different fields.

While previous studies have investigated the antioxidant and phenolic profiles of *Aronia melanocarpa* from various regions, there is a lack of data on aronia cultivated in Türkiye, particularly from the Edirne province. Unlike earlier reports, the present study systematically characterizes not only the total phenolic and flavonoid content, but also provides a detailed polyphenolic fingerprint using LC–MS/MS, elemental and heavy metal composition via ICP‐MS, and comparative antioxidant and antibacterial evaluations. To our knowledge, this is the first comprehensive assessment of Edirne‐grown aronia, offering region‐specific insights into its bioactive potential and its suitability for use in functional foods and natural antimicrobial formulations. In this regard, it was aimed to determine the total phenolic and flavonoid contents and minerals, as well as in vitro antioxidant and antibacterial activity of the juice obtained from aronia grown in Edirne province by mechanical pressing. The results obtained are important as they will provide important data on aronia juice obtained by mechanical pressing, which is becoming increasingly popular and widely used in the production of aronia juice. In addition, it was aimed to provide a comprehensive reference for the further use, development, and added value of Edirne aronia in the region, and also to reveal its advantages/disadvantages compared to species grown in other parts of the world.

## 2 Materials and Methods

### 2.1 Chemicals and Reagents

All chemicals and reagents such as Folin–Ciocalteu's phenol reagent, phenolic standards (3,4,5‐Trihydroxybenzoic acid (gallic acid, anhydrous, for synthesis), (+)‐catechin), 22‐Diphenyl‐1‐picrylhydrazyl (DPPH), 2,2′‐azinobis (3‐ethylbenzothiazoline‐6‐sulfonic acid) diammonium salt (ABTS, ≥ 98%), sodium carbonate (Na_2_CO_3_, ≥ 99.5%), sodium nitrate (NaNO_3_, ≥ 99.0%), aluminum chloride hexahydrate (AlCl_3_.6H_2_O, 99%), sodium hydroxide (NaOH, ≥ 97.0%), and potassium persulfate (K_2_S_2_O_8_, ≥ 97.0%) were purchased from Sigma Aldrich and used as received without any purification procedure. The standard gram‐negative and gram‐positive strains such as *E. coli* ATCC 25922, *P. aeruginosa* ATCC 27853, *S. aureus* ATCC 29213, and *E. faecalis* ATCC 29212 were procured from the Pharmaceutical Microbiology Laboratory of Trakya University. The antibiotics such as ciprofloxacin and gentamicin were purchased from Sigma Aldrich and used as received. Both Mueller Hinton Agar (MHA) and Mueller Hinton Broth (MHB) utilized for microbial cultures were purchased from Merck. All utilized solvents used in the analyses were HPLC grade and procured from Sigma Aldrich and used without any distillation procedure.

### 2.2 Plant Material and Sample Preparation

The commercially harvested ripe aronia was procured 15 days apart from a local farm located in Büyükdöllük Village, Edirne province (41.75234 latitude 26.59857 longitude) during October 2024 and immediately stored in a plastic bag in the laboratory refrigerator (at −20°C) until further analysis. When analyses are performed, fruits of aronia that are not yet rotten or shriveled are selected, washed, and rinsed with cold tap water, then left to dry at room temperature. Then the aronia is pressed in a hand‐operated hydraulic press machine and filtered through coarse filter paper 2 times to obtain the AFJ.

### 2.3 Total Phenolic Contents

The total phenolic content (TPC) of the obtained AFJ was determined by the well‐known Folin–Ciocalteu method using UV–Vis (Mecasys Optizen POP Series) spectroscopy. This method is based on the spectrophotometric measurement of the blue color formed as a result of the redox reaction in which the phenolic compounds in the sample reduce the Folin–Ciocalteu reagent in a basic environment and oxidize themselves (Pérez et al. 2023). In our study, the total phenolic content assay of the samples was carried out by making a series of modifications. First, 0.1 mL of AFJ diluted at a certain rate was taken into the test tubes. 4.5 mL of distilled water and 0.1 mL of Folin–Ciocalteu reagent were introduced, and the mixtures in the tubes were vortexed for 10 s; after waiting for 3 min, 0.3 mL of 2% (v/w) Na_2_CO_3_ was added to the tubes. At the end of this period, the tubes containing the mixtures were kept in a shaking water bath (Witeg‐Wisebath, WSB Series SS306) at room temperature at 125 rpm for 2 h, and then the absorbance values were measured at 765 nm with the help of a UV–Vis spectrophotometer. The blank sample to be used in zeroing the spectrophotometer was prepared under the same conditions using pure water instead of the sample. In the experiments, gallic acid solutions with different concentrations (50, 100, 200, 300, 400, and 500 μg mL^−1^) were used for preparing the standard curve. Using the same experimental procedures, the total phenolic content of the AFJ was determined as mg GAE (Gallic Acid Equivalent) mL^−1^ fresh volume (mg GAE mL^−1^ FV) with the linear equation obtained from the gallic acid concentration‐absorbance plot (50–500 μg mL^−1^, *y* = 0.0114*x* − 0.0067; *R* ^2^ = 0.9676). The results achieved from the test solutions were reported as the mean of triplicate measurements ±SD.

### 2.4 Total Flavonoid Contents

Total flavonoid content (TFC) in AFJ was determined by spectrophotometric colorimetry with minor modifications to a previously published method (Wang et al. 2022). The reaction mixture was prepared by mixing 1 mL of AFJ sample (diluted at a certain ratio) and 0.3 mL of 5% (w/v) sodium nitrate. The mixture was allowed to stand for 5 min. Then, 0.3 mL of 10% (w/v) aluminum chloride solution was added to the mixture, and 2.0 mL of 1 M sodium hydroxide solution was added (at 6. min) and mixed. Finally, 2.4 mL of distilled water was added and the tubes were mixed. The absorbance values of the samples were determined by UV–Vis spectrophotometer at 510 nm. TFC was expressed as the catechin equivalent mL^−1^ fresh volume (mg CE mL^−1^ FV) of the samples by calibration curve (50–500 μg mL^−1^, *y* = 0.0031*x* + 0.1745, *R* ^2^ = 0.972). The experiments were performed in 3 replicates, and the average value of the results was reported with ±SD.

### 2.5 Individual Phenolic Compounds

Phenolic compounds of juice obtained from aronia fruits were established by means of liquid chromatography–tandem mass spectrometry (LC–MS/MS, Agilent 6460) system with triple quadrupole mass spectrometer equipped with electrospray ionization (ESI) interface (Agilent Technologies, CA, USA). In the analysis, first, five points were prepared using certified standard compounds with concentrations of 5–10–25–50–100 ng mL^−1^ and a calibration curve was created. The appearance of compounds in a certain time interval in the chromatogram, the ratios of the main ion, and verification ions are compared with the ratios of the fragmented ions obtained from the matrix spike. The area of the peaks obtained as a result of the analysis is plotted against the concentration of the added standard in the sample. Thus, the substance is determined with high sensitivity and accuracy. Before the analysis, two different isolation processes were performed, namely hydrolysis and non‐hydrolysis (direct extraction) methods, for the separation of different compounds that may be present in the samples. The main purpose of using these methods was to separate both sugar‐containing and basic phenolic compounds from the samples. All required procedures, hydrolysis and non‐hydrolysis extraction methods were carried out as previously described (Bayram et al. 2020).

### 2.6 Elemental Composition

Inductively coupled plasma mass spectrometry (ICP‐MS, Agilent 7800, USA) was utilized for the determination of the mineral and heavy metal elements with their concentrations in the AFJ sample. ICP‐MS analysis of elemental and heavy metal content was conducted in accordance with U.S. EPA Method 200.8 (Determination of Trace Elements in Waters and Wastes by ICP‐MS) and EPA Method 200.7 for multi‐element detection in food‐related matrices. The sample preparation procedure for the ICP‐MS analysis and required experimental procedures were as follows (Bayram et al. 2020; Oroian et al. 2015): The samples (0.5 mL) were mixed with 9 mL of HNO_3_ and 1 mL of H_2_O_2_ after being placed into a Teflon digestion vessel and burned in a microwave‐assisted digestion system (Milestone, Italy). The instrument parameters were set to reach 200°C in 15 min, and the samples were additionally held at 1000 W for 15 min. The sample was filtered through a Whatman No. 1 filter paper and diluted with 50 mL of ultra‐pure water before the analysis. The mixture of HNO_3_ and H_2_O_2_ (9:1, v/v) was used as the blank sample and prepared in the same way mentioned above. The results were presented as the average of three independent measurements in mg L^−1^ FV with ±SD.

### 2.7 Radical Scavenging Capacity Assessed by Chemical Assays

#### 2.7.1 In Vitro Antioxidant Activity by DPPH ^•^ Assay

This method is based on the reduction of purple chromogen radical to hydrazine responsible for the light yellow color by proton transfer of compounds with antioxidant character and the measurement of this color change spectrophotometrically (Apak et al. 2020). In this study, the DPPH^•^ solution to be used in determining in vitro antioxidant activity was obtained by adding 0.0150 mg of DPPH^•^ dissolved with methanol to 500 mL to obtain a concentration of 30 ppm. 3.9 mL of DPPH^•^ solution and 0.1 mL of AFJ were introduced into the test tubes (dilution was done as required). The tubes were vortexed and sealed with aluminum foil and stored in the dark at room temperature for 30, 60, and 120 min. At the end of the storage periods, absorbance values were read on a UV–Vis spectrophotometer at a wavelength of 517 nm (Bahtiyar et al. 2024). The percentage antioxidant activity values obtained from the absorbance values were calculated from the Equation (1) given below and reported as the average of three independent measurements with ±SD.

(1) $$\text{Scavencing activity}\;\mathrm{on}\;\text{DPPH radical}\;\left(\%\right)=\left(\frac{A_{c}-A_{s}}{A_{c}}\right)\times 100$$

where *A*_*c*_ and *A*_*s*_ express the absorbance values of DPPH^•^ solution (without the sample) and the samples reacted with DPPH^•^ solution, respectively.

#### 2.7.2 In Vitro Antioxidant Activity by ABTS ^+•^ Assay

For antioxidant activity by ABTS^+•^ method, first, 7 mM ABTS stock solution and 2.25 mM potassium persulfate solution were mixed in equal volumes. ABTS was kept in the dark for 16 h before being applied for radical formation. Then, ABTS^+•^ solution was diluted with ethanol until it reached an absorbance of 0.7 (±0.05) at 734 nm (Re et al. 1999). ABTS radical scavenging ability of AFJ was determined by making minor modifications to the method specified by Pellegrini et al. (Orellana‐Palma et al. 2020). 1950 μL of ABTS solution was added to 50 μL of diluted AFJ sample, and the suspension was incubated at room temperature in the dark for 30 min. Ethanol was used as a blank. After incubation, the solution was measured by UV–vis spectrophotometry at 734 nm. The radical scavenging activity values were calculated using Equation (1) and reported as the average of three independent measurements with ±SD. In the specified formula, Ac gives the absorbance of the ABTS solution (without sample), and As gives the absorbance of the sample and ABTS solution mixture at the end of the incubation period. For both antioxidant activity analyses, in vitro antioxidant concentrations (IC_50_) causing 50% inhibition were read from the graph drawn with the % inhibition values calculated against samples prepared at different concentrations.

### 2.8 Antibacterial Activity Test

The antibacterial activity characteristics of the AFJ samples were evaluated against both gram‐negative (*E. coli and P. aeruginosa*) and gram‐positive ( *S. aureus* and *E. faecalis* ) by the Microdilution method through EUCAST directions (Kahlmeter et al. 2006). The bacterial isolates were subcultured in MHA plates and incubated overnight at 37°C for 24–48 h. The pure colonies were transferred to MHB for bacterial growth and were incubated in the appropriate conditions overnight. The bacterial suspensions were prepared according to McFarland 0.5 density (1 × 10^8^ CFU mL^−1^). This suspension was diluted 1:20 (5 × 10^6^ CFU mL^−1^) and a final working suspension (5 × 10^5^ CFU mL^−1^) was obtained in the wells of the microplates. The standard solutions of ciprofloxacin and gentamicin antibiotics in sterile distilled water were prepared by the guidelines of CLSI M100‐S28 and M27‐A3 and EUCAST (ISO 20776‐2:2021 2007; Kahlmeter et al. 2006; Wayne 2011). The susceptibility test was performed with MHB for bacteria. Firstly, 100 μL of MHB was added to each well. After placing 100 μL of sample in the first well, 100 μL of the mixture was transferred to the other wells in series to provide a 2‐fold dilution. After the dilutions, a 10 μL bacterial inoculum was added to each well of the microdilution trays. Then, the trays were incubated at 37°C for bacterial growth in a humid chamber, and minimum inhibitory concentration (MIC) endpoints were read after 24 h of incubation. The lowest concentration of the compound that completely inhibits macroscopic growth was determined, and minimum inhibitory concentrations (MICs) were reported. A line of negative control wells was created to observe the difference between bacterial growth sites and non‐growth sites. The pure microorganisms were used as positive control, whereas the pure media were used as negative control wells. MIC values were calculated based on the mass concentration of AFJ. Considering the density of AFJ (1 mL = 1.147 g), a 1:16 dilution corresponds to approximately 71.7 mg mL^−1^ (71,700 μg mL^−1^), and a 1:4 dilution corresponds to 286.75 mg mL^−1^ (286,750 μg mL^−1^). These concentrations were used to express MIC values in μg mL^−1^ for standardization and literature comparison (1 mL of aronia juice is equivalent to 1.147 g).

### 2.9 Statistical Analysis

Statistical analyses were performed using IBM SPSS Statistics 27 (SPSS Inc., Chicago, IL, USA). Significant differences between groups were determined using Duncan's multiple range test. One‐way analysis of variance (ANOVA) was used to assess significant differences in the % inhibition values by sample concentration and time. Differences were considered statistically significant at a 95% confidence level. All results were expressed as mean ± standard deviation (*n* = 3).

## 3 Results and Discussion

**FIGURE 1:**
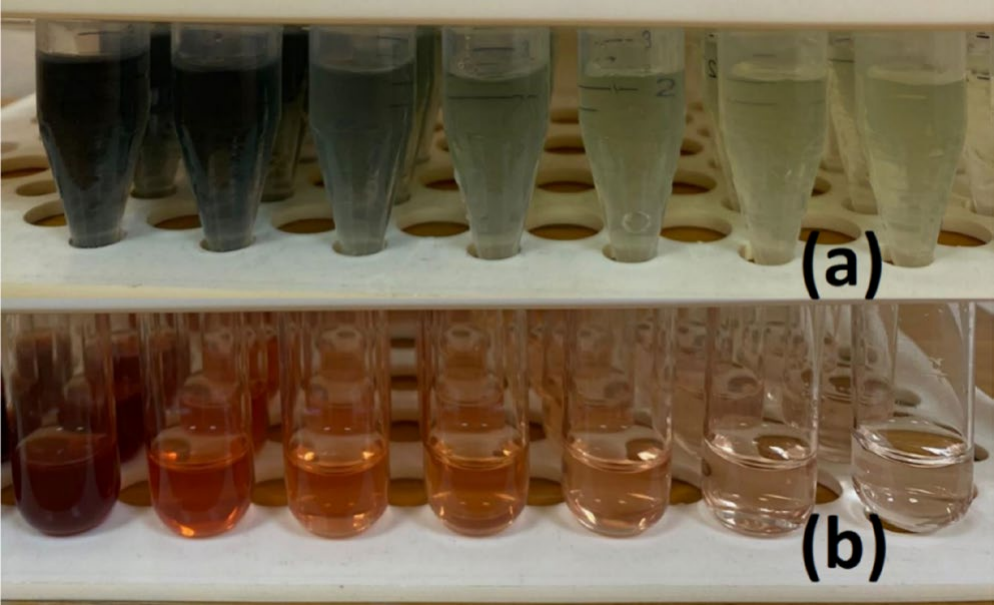
The visual appearances of the test tubes during the TPC (a) and TFC (b) tests, 50 to 2‐fold dilutions from left to right.

In recent years, aronia has attracted much attention in the food industry for the production of many different types of dietary products, especially fruit teas, jams, juices, and dietary supplements. Aronia has been referred to as a super‐fruit in the literature because it has many bioactivities that are potentially beneficial for human health (Ren et al. 2022). In this perspective, it has become necessary to examine some of the characteristic features of aronia, which were first grown in the Edirne region about 4 years ago, and to bring them to the literature. For this purpose, AFJ used in this study was isolated by cleaning, pressing, and filtering in a sequential manner and analyzed in terms of its phenolic, macro/micro‐element ingredients, as well as in vitro antioxidant and antibacterial capacities. It is well established in the literature that polyphenols are among the primary dietary sources of antioxidants (Rana et al. 2022). The substances rich in polyphenols exhibit antioxidant activity by scavenging free radicals in the environment and are ultimately potentially used in disease prevention and treatment. In this perspective, among many different types of berries, aronia stands out among others in terms of its high polyphenol content (Negreanu‐Pirjol et al. 2017). Polyphenols are basically divided into two main groups: (i) phenolic acids and (ii) flavonoids. It is well known that juices obtained from aronia berries are rich in polyphenolic compounds. Variations in fruit processing methods, juice extraction techniques, harvest timing, and ripeness levels can significantly influence the phenolic content of fruit products (Mayer‐Miebach et al. 2012). Meanwhile, aronia products, including fruit juices, remain rich in phenolic compounds and therefore continue to have high antioxidant activity (Tolić et al. 2015). Considering the TPC and TFC in AFJ prepared here by mechanical pressing, these values were determined to be 2.37 ± 0.28 mg GAE mL^−1^ FV by using the Folin–Ciocalteu method and 4.68 ± 0.45 mg CE mL^−1^ FV, respectively. The visual appearances of the color changes during the TPC and TFC tests were illustrated in Figure 1a,b.

On the other hand, polyphenolic compounds of AFJ were determined separately by LC–MS/MS in terms of major fragment ion versus retention time. Table 1 represents the concentration of the polyphenolic compounds (mg L^−1^ FW) with their MRM transitions (*m/z*) and retention time (rt, min) of non‐hydrolyzed/hydrolyzed AFJ. As can be clearly seen from Table 1, ten of the eighteen phenolic compounds found in AFJ were phenolic acids, while eight of them were flavonoids. Regarding the phenolic acid levels of the non‐hydrolyzed sample, the highest concentration in AFJ belonged to Chlorogenic acid (5′‐caffeoyl quinic acid) with a value of 2075.19 ± 14.94 mg L^−1^ FV as good agreement with the literature (Go et al. 2024). Moreover, the most abundant flavonoid in the same sample was determined to be Rutin with a concentration of 24.66 ± 0.82 mg L^−1^ FV. Considering the hydrolyzed sample, Chlorogenic acid is the most common ingredient again in AFJ with a concentration of 640.58 ± 41.36 mg L^−1^ FV, whereas Quercetin was the major flavonoid detected and its concentration was determined to be 25.50 ± 0.81 mg L^−1^ FV. As can be seen from the relevant data, the most common and abundant polyphenolic compounds of AFJ were Chlorogenic acid, Rutin, Caffeic acid, and Quercetin. In this context, the mass spectrum of Chlorogenic acid presented a molecular ion [M‐H]^−^ at *m*/*z* 353, and its MS/MS spectrum showed characteristic fragment ions at *m*/*z* 191. Moreover, the mass spectrum of Rutin presented a molecular ion [M‐H]^−^ at *m*/*z* 609, and its MS/MS spectrum showed characteristic fragment ions at *m*/*z* 300. The molecular ion [M‐H]^−^ at *m*/*z* 179 and characteristic fragment ions at *m*/*z* 135 and 117 referred to Caffeic acid; these values were [M‐H]^−^ at *m*/*z* 300 and *m*/*z* 179 and 151, respectively, for Quercetin. The reason why the total concentration of phenolic acids obtained in the hydrolyzed sample was higher than that of the non‐hydrolyzed sample was due to the breaking of glycosidic bonds resulting from acidolysis (Acosta‐Estrada et al. 2014). Our results showed that AFJ is extremely rich in o‐diphenolics such as caffeic acids, epicatechin, and quercetin derivatives. These compounds have an enhancing effect on the antioxidant role due to the o‐dihydroxy structure in the B ring, which provides stability to radicals and participates in electron delocalization (Rice‐evans et al. 1995). The authors of the previous papers pointed out that the differences in the content of polyphenols of the same type of plants grown in different regions were related to seasonal variations of plant growing conditions, including temperature, exposure to the sun, rainfalls, etc. (Cohen and Kennedy 2010; de Medeiros Gomes et al. 2021).

**TABLE 1:** The polyphenolic compounds, MRM transitions, retention times, and phenolic concentrations of the AFJ with ±SD.

Polyphenolic compounds	MRM transitions (*m*/*z*)	Non‐hydrolyzed		Hydrolyzed	
		Rt (min)	Concentration (mg L^−1^ FV)	Rt (min)	Concentration (mg L^−1^ FV)
Phenolic acids					
Gallic acid	79→169	1.761	0.022 ± 0.00	1.736	1.68 ± 0.06
Protocatechuic acid	91→153	1.822	2.00 ± 0.05	1.855	25.99 ± 0.19
2.5‐dihydroxybenzoic acid	53→153	1.825	<loq	2.100	4.00 ± 0.15
Caffeic acid	117→179	3.632	0.38 ± 0.03	3.649	168.46 ± 15.53
Chlorogenic acid	82→353	3.695	2075.19 ± 14.94	3.661	640.58 ± 41.36
*p*‐coumaric acid	93→163	3.937	<loq	3.988	3.19 ± 0.14
Salicylic acid	65→137	3.726	<log	3.709	0.94 ± 0.06
Trans‐ferrulic acid	178→193	3.936	<loq	4.038	9.33 ± 1.05
Abscisic acid	219→263	4.282	0.91 ± 0.08	4.282	2.93 ± 0.10
Flavonoids					
Quercetin	179→301	4.229	4.10 ± 0.24	4.238	25.50 ± 0.81
Lutolein	151→285	4.272	0.12 ± 0.07	4.272	0.54 ± 0.37
Rutin	271→609	3.938	24.66 ± 0.82	3.946	0.37 ± 0.08
Naringenin	271→119	4.006	<loq	4.315	0.48 ± 0.09
Isorhamnetin	151→315	4.297	<loq	4.348	3.87 ± 0.06
Jaceosidin	299→329	4.406	1.42 ± 0.91	4.398	2.03 ± 1.59
Catechin+Epicatechin	179→305	3.891	4.66 ± 0.23	3.865	<loq
Gallocatechin+Epigallocatechin	179→305	3.806	10.82 ± 0.54	3.823	<log
Hesperidin	301→609	3.963	8.04 ± 0.03	3.921	0.18 ± 0.02
Phlorizin	167→435	4.075	1.38 ± 0.10	4.075	<loq

Abbreviation: loq, limit of quantification.

As with every fruit, the mineral and heavy metal content in Aronia berries may vary depending on the soil composition, agricultural practices, harvest date, and vegetation period. The minerals and heavy metals, as well as their concentrations, were determined by ICP‐MS in AFJ; the results were indicated in Table 2. The most common major minerals in AFJ were detected as K, Mg, Ca, and Na. Furthermore, quantitatively, the most prominent mineral was K, with the average concentration of 1,645.59 ± 51.53 mg L^−1^ FV, followed by the abundant Mg (average 96.65 ± 8.08 mg L^−1^ FV), Ca (90.84 ± 2.14 mg L^−1^ FV), and Na (89.90 ± 11.95 mg L^−1^ FV) minerals, respectively, indicating good agreement with corresponding results from previous studies (Kaličanin et al. 2022; Pop et al. 2022). On the other hand, it is widely accepted by scientists and the medical community that the quality of fruit products decreases with increasing concentration of environmental pollutants (especially pesticides), toxic compounds, and heavy metals (Smichowski and Londonio 2018). In this context, the chemical composition of plants commonly utilized in human nutrition or in the preparation of herbal preparations must be constantly controlled. In particular, some metals are important for the development of a plant, but, on the other hand, if they exceed a certain level, they have negative consequences for consumers.

**TABLE 2:** Macro‐ and micro‐ mineral/heavy metal contents in AFJ.

Elements	Concentration (mg.L^−1^ FV)
Minerals	
Potassium (K)	1645.59 ± 51.53
Magnesium (Mg)	96.65 ± 8.08
Calcium (Ca)	90.84 ± 2.14
Sodium (Na)	89.90 ± 11.95
Boron (B)	4.67 ± 0.22
Heavy metals	
Tin (Sn)	7.94 ± 1.01
Iron (Fe)	5.64 ± 0.60
Manganese (Mn)	3.91 ± 0.05
Nickel (Ni)	2.06 ± 0.21
Aluminum (Al)	1.86 ± 1.03
Strontium (Sr)	0.88 ± 0.01
Barium (Ba)	0.73 ± 0.16
Titanium (Ti)	0.32 ± 0.00
Copper (Cu)	0.28 ± 0.00
Lead (Pb)	0.09 ± 0.01
Chromium (Cr)	0.08 ± 0.05
Cadmium (Cd)	0.05 ± 0.01
Cobalt (Co)	0.01 ± 0.00
Molybdenum (Mo)	0.01 ± 0.00
Vanadium (V)	0.01 ± 0.00
Selenium (Se)	< 0.00
Antimony (Sb)	< 0.00
Zinc (Zn)	< 0.00
Bismuth (Bi)	< 0.00

The presence of heavy metals such as Pb, Cd, Hg, and As in plants can be attributed to environmental factors, production process, and storage conditions and may have toxic effects on humans as they contaminate plants (Campos et al. 2009; Grumezescu and Holban 2017). The results of the monitoring studies carried out on the juice obtained from aronia grown in Edirne showed that Pb and Cd levels of aronia juice were determined as 0.09 ± 0.01 and 0.05 ± 0.01 mg L^−1^, respectively. It was found that the Pb level was lower than the limit value given for berries and other small fruits in the Alimentarius codex (0.1 mg kg^−1^). There is no Cd limit value specified for fruits in the Alimentarius codex, and limit values for some foods are given in the range of 0.05–0.2 mg kg^−1^ (FAO‐WHO 2023). In this study, the heavy metal contents were constituted by Sn, Fe, Mn, Ni, and Al with the concentrations of 7.94 ± 1.01, 5.64 ± 0.60, 3.91 ± 0.05, 2.06 ± 0.2, and 1.86 ± 1.03 mg L^−1^ FV, respectively (Pop et al. 2022). Iwegbue et al. (2008) reported the concentration of Ni in six brands of fruit juices from Nigeria was measured as 0.13–7.93 (mg kg^−1^). Fathabad et al. (2018) reported the concentration range of Al and Sn as 340.62 (65.17–1039.2) and 72.33 (49.76–119.4) μg kg^−1^, respectively, in 36 branded fruit juice samples obtained from Iranian supermarkets (Fathabad et al. 2018). The Alimentarius codex (FAO‐WHO 2023) does not provide limit values for Al and Ni in fruit juices. However, the limit value for Sn is reported as 50–250 mg kg^−1^ for canned and cooked foods. In addition, while the Turkish Food Codex contaminants circular does not specify the limit value for Sn, Al, and Ni for fruit juices, the Sn limit value for beverages sold in cans is set at 100 mg kg^−1^ (Turkish Food Codex 2023).

The presence of low amounts of heavy metals such as Sn (ICP‐MS could not distinguish between organic and inorganic forms of tin (Lehmler et al. 2018)), Fe, Co, Cu, Ni, Mn, Mo, Zn, etc. in the environment and diet is essential for human health (Fernandez‐Luqueno et al. 2013; Nagajyoti et al. 2010). As a result, it has been determined that these levels of heavy metals in the juice obtained from Edirne aronia will not cause negative consequences for human health.

**FIGURE 2:**
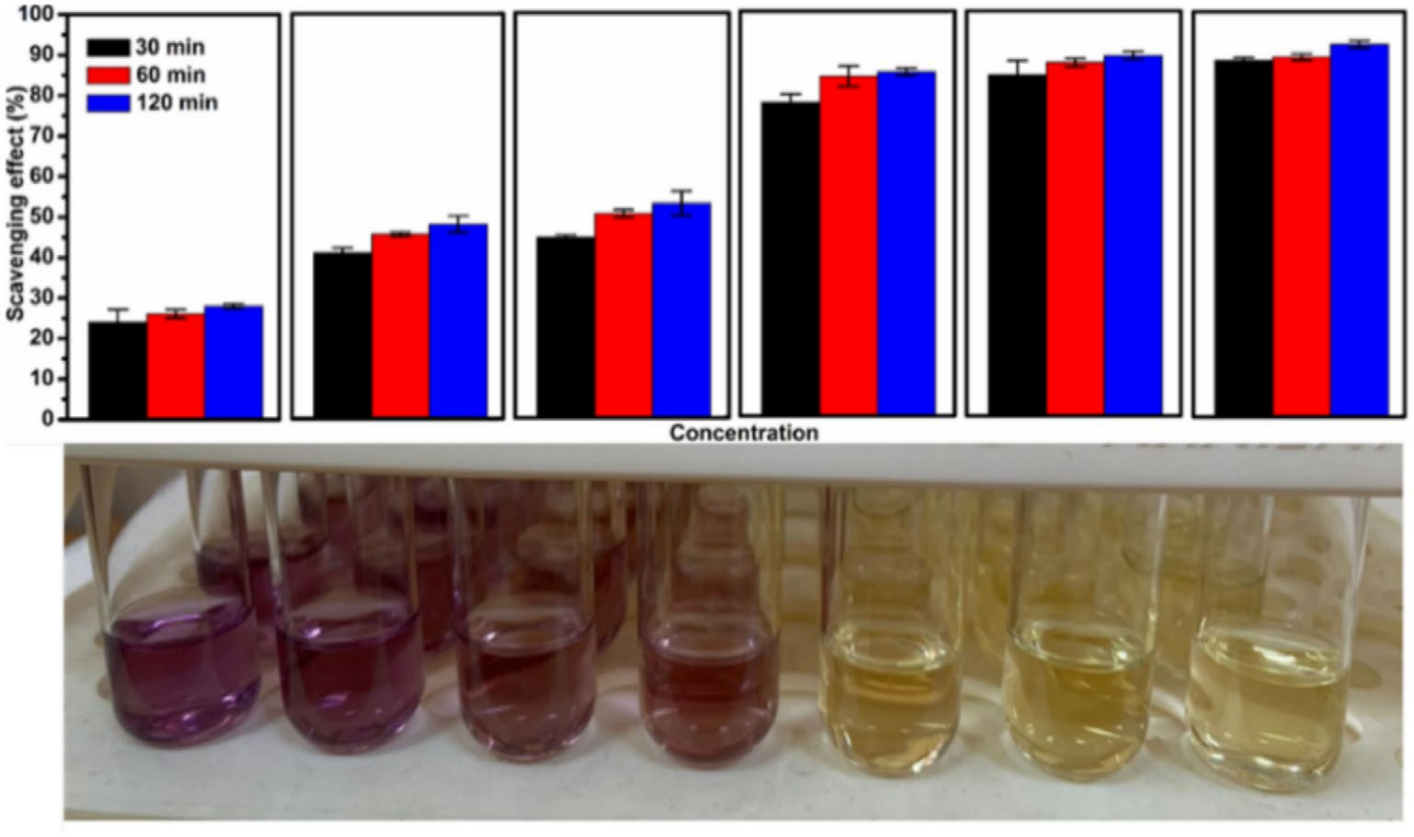
Total antioxidant activity of AFJ measured by DPPH^•^ assay and visual aspects of color changes: Effect of concentration and treatment time on antioxidant activity (concentrations were 10, 20, 25, 50, 100, and 200 μL mL^−1^ from left to right). (A–C) Different uppercase letters in the same concentration indicate statistically significant differences (*p* < 0.05). (a–e) Different lowercase letters in the same color indicate statistically significant differences (*p* < 0.05).

As mentioned earlier, Aronia is a plant rich in the amount of polyphenolic compounds that reveal numerous beneficial properties for human health. Polyphenols have multiple hydroxyl functional groups that have the ability to bind and therefore eliminate free radicals. Due to this reason, they also have a braking effect against many negativities that affect human health with its antioxidant activities (Kobus et al. 2019). The results presented in Figure 2 indicated inhibition % plots of AFJ determined by DPPH^•^ assay with concentration and treatment time variations ranging from 10 to 200 μL mL^−1^ and 30 to 120 min, respectively. It was found that DPPH% inhibition values determined at the 30th min at concentrations of 20, 25, 50 and 200 μg mL^−1^ were statistically lower than the values obtained at other times (*p* < 0.05). While the difference between the DPPH% inhibition values determined at the 60th and 120th mins at concentrations of 10, 25, 50 and 100 μg mL^−1^ was found to be insignificant (*p* > 0.05), DPPH% inhibition values at the 120th min at concentrations of 20 and 200 μg mL^−1^ were found to be statistically higher than the values obtained at other times (*p* < 0.05). At the end of the 120‐min incubation, DPPH% inhibition values showed a linear change depending on the concentration (*p* < 0.05), but no significant increase in DPPH% inhibition values was observed at the 30th and 60th mins after the 100 μg mL^−1^ concentration (*p* > 0.05). An increasing trend in inhibition % was observed with the increase in the AFJ concentration and treatment time. For the sample with a concentration of 10 μL mL^−1^, the percent inhibition values at 30, 60 and 90 min were approximately 24%, 26% and 28%, while these values were found to be 87, 89 and 92 when the concentration was 200 μL mL^−1^. Furthermore, at this stage of the study, the concentration of AFJ required to inhibit DPPH^•^ activity by 50% (IC_50_) was determined and IC_50_ level of AFJ was calculated as 26.31 ± 0.65 μL mL^−1^. The visible color change from purple to yellow in the DPPH^•^ solution, corresponding to increasing AFJ concentration, further confirmed the enhancement in antioxidant activity.

**FIGURE 3:**
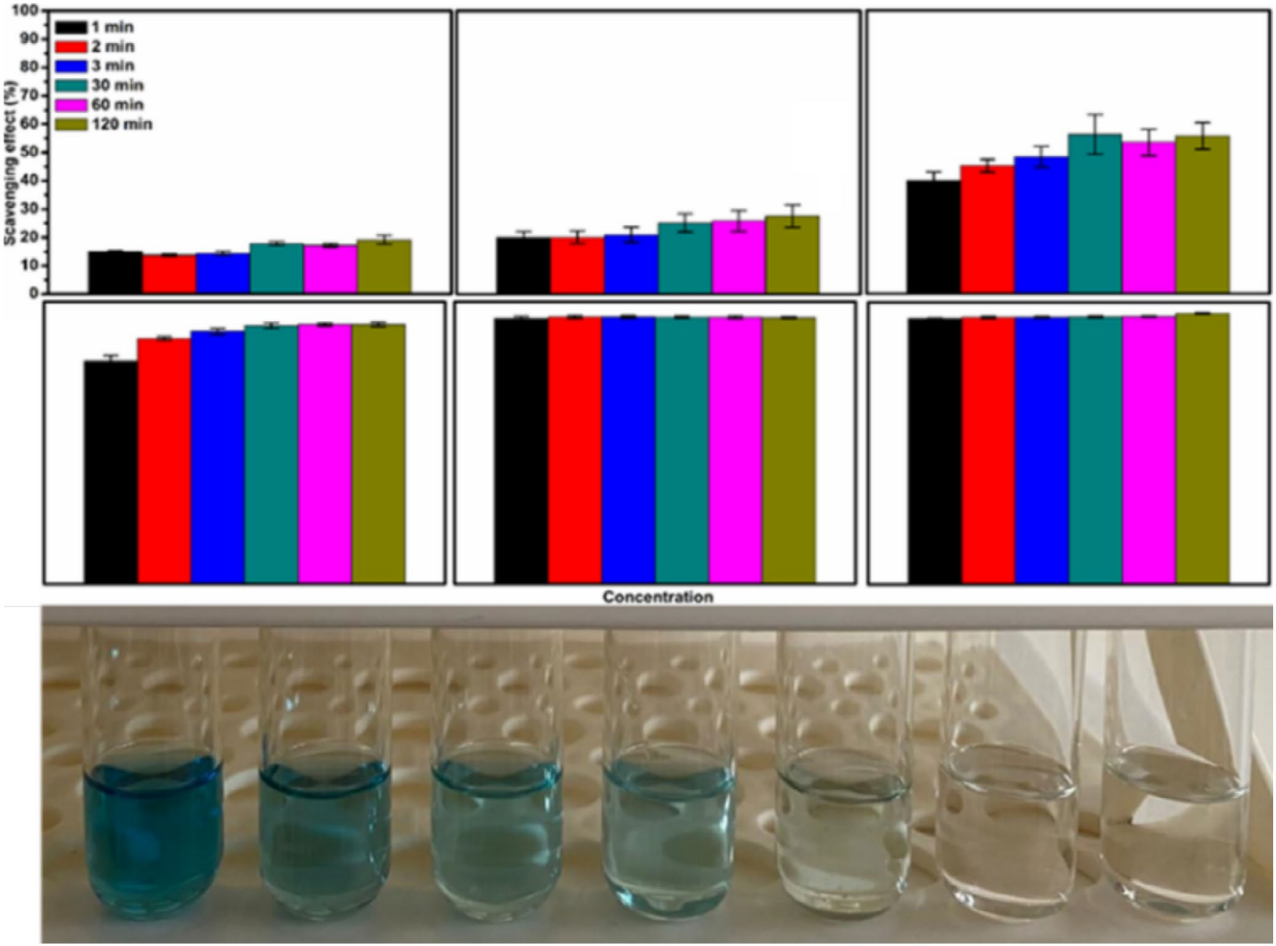
Total antioxidant activity of AFJ measured by ABTS^+•^ assay and visual aspects of color changes: Effect of concentration and treatment time on antioxidant activity (concentrations were 10, 20, 25, 50, 100, and 200 μL mL^−1^ from left to right). (A–D) Different uppercase letters in the same concentration indicate statistically significant differences (*p* < 0.05). (a–e) Different lowercase letters in the same color indicate statistically significant differences (*p* < 0.05).

At the end of the 1st, 4th, and 6th incubations, the ABTS% inhibition values obtained at the 100 and 200 μg mL^−1^ concentrations were statistically similar (*p* > 0.05). At concentrations of 50 μg mL^−1^ and above, the effect of incubation longer than 30 min on ABTS% inhibition was found to be insignificant (*p* > 0.05). Many studies have reported that ABTS and DPPH% inhibition changes are nonlinear and depend primarily on phenolic protection and incubation data. In the case of inhibition % results obtained with the ABTS^+•^ assay (Figure 3), a similar order from lowest to highest was presented, concluding that the percentage of inhibition reached its maximum at the concentration of 50 μL mL^−1^ almost regardless of the time. The IC_50_ level of AFJ was calculated as 27.99 ± 0.78 μL mL^−1^ in ABTS^+•^ assay. The color changes from green to yellow during the tests verified the increasing antioxidant activity trend. Meanwhile, it can be seen that the AFJ samples presented a very fast scavenging activity against ABTS^+•^ compared to DPPH^•^ at the same concentrations and experimental conditions. The observed differences between the two assays may be related to the various reaction mechanisms followed in the tests or to the number and position of active hydroxyl groups, double bonds, and aromatic rings whose effects may overlap with each other (Solaberrieta et al. 2020). Although specific time periods are commonly used in the literature for the deactivation of ABTS and DPPH radicals, many studies have reported that ABTS and DPPH% inhibition changes are nonlinear and depend primarily on the phenolic composition and incubation conditions (Macharáčková et al. 2021; Suhag et al. 2025). With the exception of rapidly reacting substances present in the sample (ascorbic acid, Trolox), more reactive phenolic compounds, peptides, and amino acids may require longer times to reach equilibrium. However, longer incubation times have been reported to result in more minimal increases (Macharáčková et al. 2021).

While DPPH^•^ and ABTS^+•^ assays are useful for evaluating free radical scavenging properties, they are limited to chemical interactions and do not necessarily reflect in vivo antioxidant efficacy. Therefore, additional biological assays, such as cell‐based oxidative stress models, would be needed to fully validate the health‐promoting potential of AFJ.

To support the novelty of *Aronia melanocarpa* cultivated in Edirne, a comparison with previous studies from other regions (Croatia, Serbia, Slovakia) was conducted. As seen in Table 3, the TPC in Edirne aronia juice surpasses that of Croatian samples (1.55–2.04 mg GAE mL^−1^) as reported by Tolić et al. (2015). The chlorogenic acid level in Edirne juice is over twice the average reported in Croatian samples (421–992 mg L^−1^) and significantly higher than values from Slovakia (645–840 mg L^−1^) (Lidiková et al. 2025). Similarly, key flavonoids such as rutin and quercetin are markedly elevated in the Edirne sample. Regarding mineral composition, potassium levels in Edirne juice are higher than those in juices analyzed by Tolić et al. and even exceed the total potassium content of fruits from Serbian aronia cultivars (1.47%–1.86% FW) (Radanović et al. 2012). Moreover, the IC_50_ values from DPPH^•^ and ABTS^+•^ assays demonstrate a stronger antioxidant potential compared to published juice data from Croatia. These findings confirm that the aronia fruit cultivated in Edirne possesses a distinctive and enhanced phytochemical and mineral profile, likely attributed to the unique agro‐climatic conditions of the Thrace region. Thus, this work contributes new and region‐specific evidence supporting the functional food potential of aronia from Türkiye.

**TABLE 3:** Comparative composition of *Aronia melanocarpa* juice from Edirne and other regions.

Parameter	Edirne, Türkiye (this study)	Croatia	Serbia	Slovakia
TPC	2.37 ± 0.28 mg GAE mL^−1^ juice	1.55–2.04 mg GAE mL^−1^ juice	—	8.6–13.2 g GAE kg^−1^ fruit
TFC	4.68 ± 0.45 mg CE mL^−1^ juice	Not reported	—	—
Chlorogenic acid	2075.19 ± 14.94 mg L^−1^	421.1–991.7 mg L^−1^ juice	—	645–840 mg L^−1^
Rutin	24.66 ± 0.82 mg L^−1^	4.75–17.3 mg L^−1^	—	10.2–16.1 mg L^−1^
Quercetin	25.50 ± 0.81 mg L^−1^	2.8–7.9 mg L^−1^	—	12.7–15.8 mg L^−1^
K	1645.59 ± 51.53 mg L^−1^	870–1340 mg L^−1^ (juice)	1.47%–1.86% FW (fruit)	—
Ca	90.84 ± 2.14 mg L^−1^	63–89 mg L^−1^	0.43%–0.49% FW	—
Mg	96.65 ± 8.08 mg L^−1^	52–73 mg L^−1^	—	—
IC_50_ (DPPH^•^)	26.31 ± 0.65 μL mL^−1^	~30–36 μL mL^−1^	—	—
IC_50_ (ABTS^+^)	27.99 ± 0.78 μL mL^−1^	~31–35 μL mL^−1^	—	—

**FIGURE 4:**
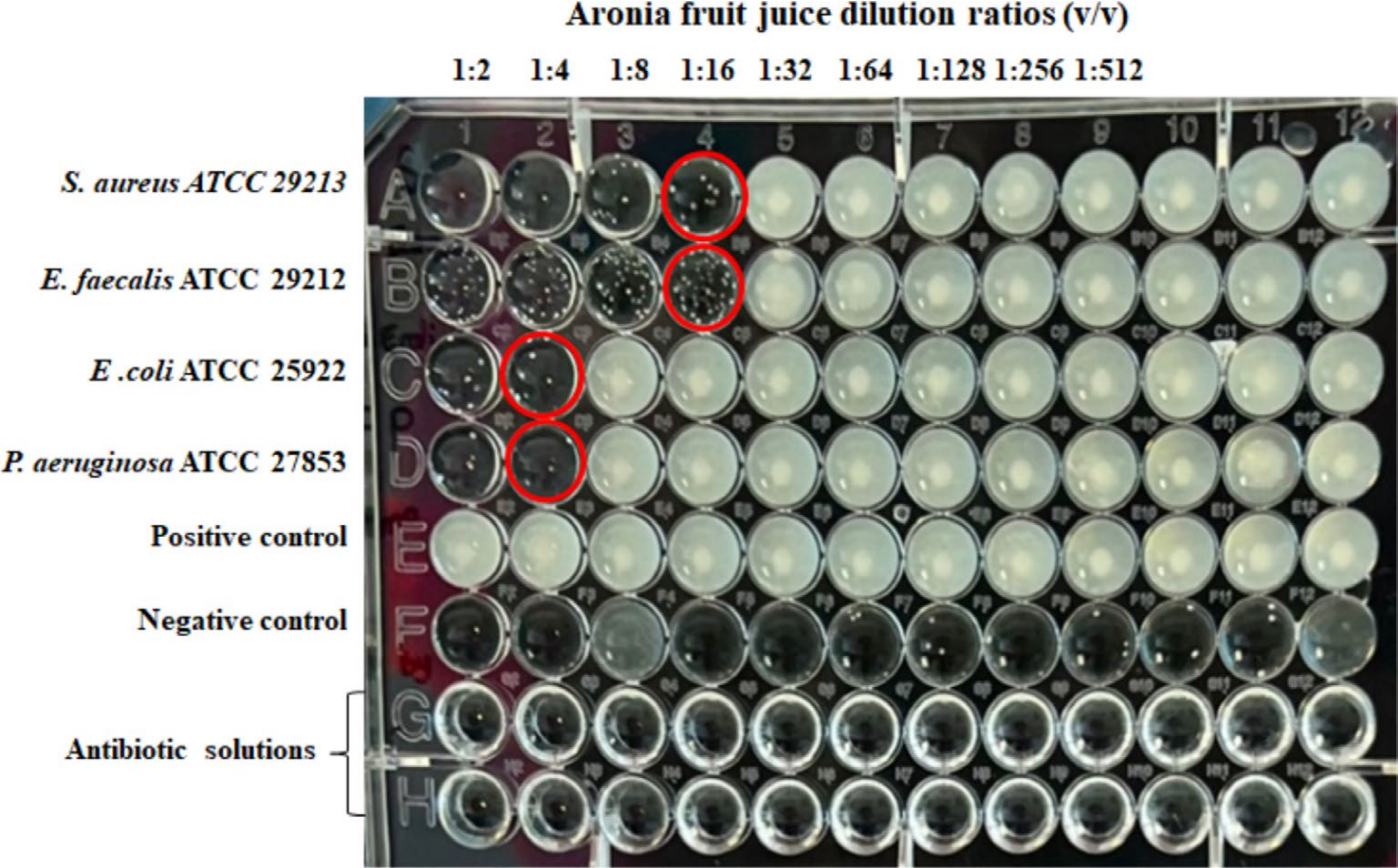
Visual appearances of turbidity of the bacteria‐inoculated, positive and negative control, and antibiotic solution samples in wells after incubation.

The antibacterial activity of juices obtained from plants such as blackcurrant, cranberry, raspberry, and aronia on common pathogenic Gram‐negative and Gram‐positive bacteria has been the subject of many research and development studies (Denev et al. 2019; Kranz et al. 2020). In literature studies, it has been discovered that fruit phytochemicals reduce pathogenicity by directly or indirectly inhibiting bacterial growth (Suriyaprom et al. 2022). Therefore, the research to obtain new antimicrobial compounds from natural sources continues at an increasing pace (Parekh et al. 2005). In our study, the juice obtained by pressing the analyzed Edirne aronia showed various antimicrobial activities against the tested bacterial strains, occurring with minimum inhibitory concentrations (Table 4). As can be seen from the MIC values and visual turbidity appearances (Figure 4), the results distinctly indicated that higher antibacterial activity was achieved against Gram‐positive strains compared to that of Gram‐negative strains. The MIC value against to gram‐positive bacteria was obtained at a dilution rate of 1:16 (286,750 μg mL^−1^), whereas this value was determined to be 1:4 (71,700 μg mL^−1^) against gram‐negative bacteria. This observation may be due to the difference in the structure of the cell structure the outer membrane of these bacterial species, which makes Gram‐positive species more sensitive to the effects of compounds found in AFJ in agreement with the results obtained by Puupponen‐Pimiä et al. (2001). Compared to standard antibiotics used as positive controls (gentamicin, ciprofloxacin), which typically inhibit bacterial growth at MICs of 0.5–2 μg mL^−1^, the AFJ exhibited substantially higher MIC values. Thus, while showing notable activity, the AFJ's efficacy is markedly lower than conventional antibiotics and should be considered as a supplementary, not standalone, antibacterial agent.

**TABLE 4:** Minimum inhibition concentrations of AFJ.

AFJ (μg mL^−1^)	*S. aureus* ATCC 29213	*E. faecalis* ATCC 29212	*E. coli* ATCC 25922	*P. aeruginosa* ATCC 27853
573,500	−	−	−	−
286,750	−	−	−	−
143,375	−	−	+	+
71,687	−	−	+	+
35,850	+	+	+	+
Gentamicin	−	−	−	−
Ciprofloxacin	−	−	−	−

*Note:* The MIC values of Gentamicin and Ciprofloxacin used as standard antimicrobial agents were determined as 1 μg mL^−1^ and 0.5 μg mL^−1^, respectively.

Abbreviations: −, no growth; +, growth.

## 4 Conclusion

The worldwide popularity of aronia is increasing day by day not only due to its nutritional values but also because of its unique effects on human health. This study is the comprehensive investigation of the phytochemical, elemental, antioxidant, and antibacterial profile of *Aronia melanocarpa* cultivated in the Edirne region of Türkiye. A key innovation is the discovery of high concentrations of chlorogenic acid and key flavonoids such as rutin and quercetin in the Edirne‐grown samples, surpassing levels reported in some other regions. This highlights the potential of Thrace's agro‐climatic conditions to enhance functional properties of aronia. Additionally, antibacterial activity was correlated with TPC; it was concluded that AFJ achieved in this study exhibited measurable antibacterial activity, particularly against Gram‐positive strains, although much higher concentrations were required compared to standard antibiotics. This suggests a potential role in functional food applications rather than clinical antimicrobial use. Despite these promising findings, gaps remain in the understanding of in vivo bioactivity, long‐term safety, and the bioavailability of these compounds. Future research should focus on clinical validation, formulation strategies to enhance stability and absorption, and exploration of synergistic effects with other bioactives. Moreover, future studies should employ cell‐based oxidative stress models, microbial biofilm assays, and animal models to assess bioactivity under physiologically relevant conditions. Additionally, metabolomic profiling, bioavailability studies, and pharmacokinetic modeling are essential to understand how phenolic compounds are absorbed, metabolized, and exert biological effects in vivo. From a broader perspective, this study supports the use of Edirne‐grown aronia as a locally‐sourced, health‐promoting ingredient in nutraceuticals, functional beverages, or clean‐label food preservatives. These findings contribute not only region‐specific data but also open new avenues for product development and health‐focused applications of this underutilized superfruit.

## Author Contributions

**Fatmagul Halici Demir:** conceptualization (equal), data curation (equal), formal analysis (equal), funding acquisition (equal), investigation (equal), methodology (equal), writing‐review and editing (equal). **Gokhan Acik:** conceptualization (equal), data curation (equal), formal analysis (equal), funding acquisition (equal), investigation (equal), methodology (equal), supervision (lead), writing – original draft (lead), writing – review and editing (lead). **Duygu Sahin Gul:** data curation (equal), formal analysis (equal), funding acquisition (equal), methodology (equal).

## Ethics Statement

This study did not contain any research activities involving animals or human participants performed by any of the authors.

## Conflicts of Interest

The authors declare no conflicts of interest.

## Supporting information


**Data S1:** fsn370784‐sup‐0001‐supinfo.xlsx.

## Data Availability

The data that support the findings of this study are available from the corresponding author upon reasonable request.
