# Correction to “The Bioactive Potential of Fruit Juice of Black Chokeberry (*Aronia melanocarpa*) Produced in Edirne Province‐Türkiye: Phenolic Profile, Elemental Composition, and In Vitro Antioxidant and Antibacterial Activities”

**DOI:** 10.1002/fsn3.71404

**Published:** 2025-12-30

Halici Demir F, Acik G, Sahin Gul D. The Bioactive Potential of Fruit Juice of Black Chokeberry (Aronia melanocarpa) Produced in Edirne Province‐Türkiye: Phenolic Profile, Elemental Composition, and In Vitro Antioxidant and Antibacterial Activities. *Food Sci. Nutr.* 2025;13(9):e70784. https://doi.org/10.1002/fsn3.70784

In Figures 2 and 3, the letters above the error bars are missing. The descriptions of the letters are in the figure caption. The corrected figures appear below.

**FIGURE 2:**
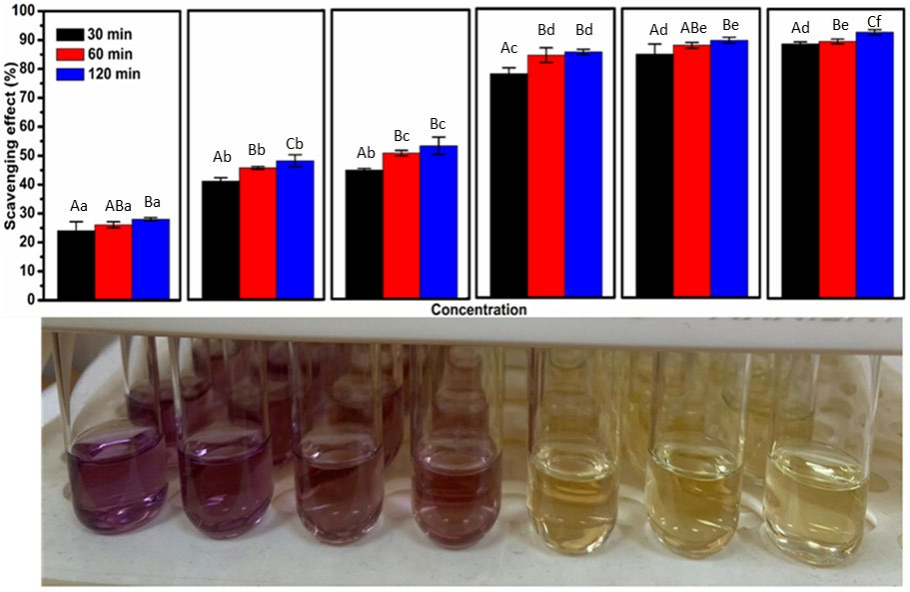
Total antioxidant activity of AFJ measured by DPPH^•^ assay and visual aspects of color changes: Effect of concentration and treatment time on antioxidant activity (concentrations were 10, 20, 25, 50, 100, and 200 μL.mL^−1^ from left to right). *A–C: Different uppercase letters in the same concentration indicate statistically significant differences (*p* < 0.05). a–e: Different lowercase letters in the same color indicate statistically significant differences (*p* < 0.05).

**FIGURE 3:**
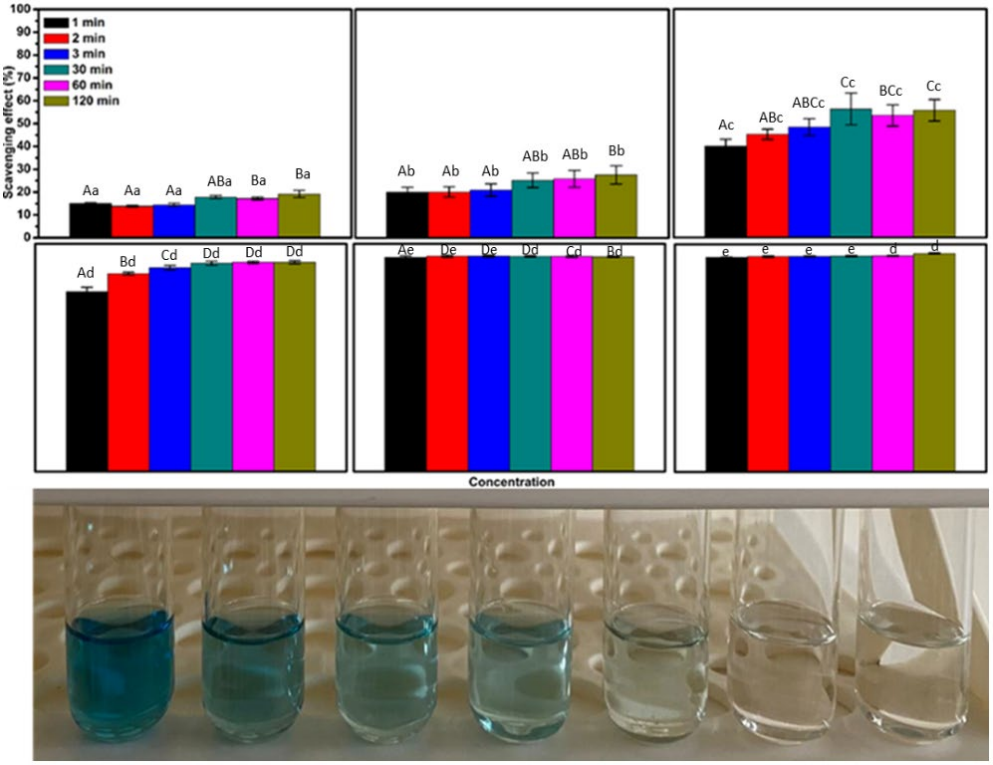
Total antioxidant activity of AFJ measured by ABTS^+•^ assay and visual aspects of color changes: Effect of concentration and treatment time on antioxidant activity (concentrations were 10, 20, 25, 50, 100, and 200 μL.mL^−1^ from left to right). *A–D: Different uppercase letters in the same concentration indicate statistically significant differences (*p* < 0.05). a–e: Different lowercase letters in the same color indicate statistically significant differences (*p* < 0.05).

We apologize for this error.

